# A critical appraisal of phloem-mobile signals involved in tuber induction

**DOI:** 10.3389/fpls.2013.00253

**Published:** 2013-07-16

**Authors:** Paula Suárez-López

**Affiliations:** Molecular Genetics Department, Centre for Research in Agricultural Genomics, CSIC - IRTA - UAB - UBBarcelona, Spain

**Keywords:** tuberization, potato, long-distance signaling, FLOWERING LOCUS T, gibberellins

## Abstract

The identification of FLOWERING LOCUS T (FT) and several FT homologs as phloem-mobile proteins that regulate flowering has sparked the search for additional homologs involved in the long-distance regulation of other developmental processes. Given that flowering and tuber induction share regulatory pathways, the quest for long-distance tuberization signals has been further stimulated. Several tuberization regulators have been proposed as mobile molecules, including the FT family protein StSP6A, the plant growth regulators gibberellins and the microRNA miR172. Although some of these hypotheses are attractive and plausible, evidence that these molecules are transmissible in potato has yet to be obtained. Two mRNAs encoding transcription factors, StBEL5 and POTATO HOMEOBOX 1 (POTH1), are mobile and correlate with tuber induction. However, evidence that StBEL5 or POTH1 are required for tuberization is not available yet. Therefore, there are several good candidates for long-distance molecules in the tuberization process. Further research should test their role as systemic tuberization signals.

The induction of tuber formation is a key developmental transition for the production of potatoes, one of the most important food crops. Understanding the regulation of tuber induction is essential to devise strategies to improve tuber yield and quality. During the last two decades we have started to comprehend this regulation, with the identification of genes that control tuberization (Jackson, 1999; Abelenda et al., 2011). This has been facilitated by the tremendous progress in understanding the control of flowering, which is similar to tuberization in aspects such as the response to photoperiod and the involvement of phloem-mobile signals (Suárez-López, 2005; Abelenda et al., 2011). This Perspective paper focuses on recent findings that suggest several molecules as candidates for systemic signals controlling tuber induction.

## LONG-DISTANCE SIGNALS REGULATE TUBERIZATION AND FLOWERING

Short day (SD) photoperiods promote tuberization, whereas long days (LDs), high nitrogen levels and high temperatures inhibit or delay tuberization. Within the tuberization process, it is important to distinguish between tuber induction and tuber development and growth. Induction takes place when signals are produced in leaves and transported through the phloem to underground stems (stolons), or when mobile signals that inhibit tuberization are repressed (Jackson, 1999; Suárez-López, 2005). This leads to the initiation of tuber development and growth, which determines tuber shape, number, and weight. Although tuber yield is often used to assess tuber induction, changes in tuber yield can result from alterations in many different factors, including overall plant growth, photoassimilate partitioning, the strength of induction, tuber development, etc. (Ewing and Struik, 1992). The time of tuber initiation is therefore a much better indicator of tuber induction than tuber yield.

Grafting experiments using potato plants induced and non-induced to tuberize demonstrated the existence of transmissible substances decades ago (Gregory, 1956; Chapman, 1958), but the identification of these signals has proven difficult. Recent advances in the study of other developmental processes provide hints for finding long-distance tuberization signals. The intensive search for a phloem-mobile flowering signal, called florigen, has led to the identification of several FLOWERING LOCUS T (FT) family members as leaf-produced proteins that travel to the shoot apical meristem, where they induce flowering (Turck et al., 2008; Tsuji et al., 2013). In *Arabidopsis thaliana*, *FT* expression is activated by the transcriptional regulator CONSTANS (CO) in leaf phloem cells in response to floral inductive photoperiods (An et al., 2004; Ayre and Turgeon, 2004).

However, florigen is not a single molecule. Positive and negative transmissible regulators of flowering exist (Bernier, 1988; Matsoukas et al., 2012). Several FT family members can perform these functions. In rice, Heading date 3a (Hd3a) and RICE FLOWERING LOCUS T 1 (RFT1) act as florigenic signals under different photoperiods (Tamaki et al., 2007; Komiya et al., 2009). In Arabidopsis ATC acts as a mobile repressor or antiflorigen and TWIN SISTER OF FT (TSF) might function as a florigen (Yamaguchi et al., 2005; Mathieu et al., 2007; Jang et al., 2009; D’Aloia et al., 2011; Huang et al., 2012). In addition, several FT-related proteins have been detected in phloem exudates of diverse species (Giavalisco et al., 2006; Lin et al., 2007; Aki et al., 2008).

Many RNAs are present in phloem exudates and a few have been reported to act in long-distance signaling (Sasaki et al., 1998; Ruiz-Medrano et al., 1999; Kim et al., 2001; Doering-Saad et al., 2002; Haywood et al., 2005). Movement of *FT* and *ATC* RNAs has been shown, but the *FT* RNA accelerates flowering less effectively than the protein and the RNA together (Li et al., 2009, 2011; Huang et al., 2012; Lu et al., 2012). Other reports indicate that translocation of the FT protein, but not the RNA, is required to promote flowering (Lifschitz et al., 2006; Mathieu et al., 2007; Notaguchi et al., 2008). These findings suggest that movement of the *FT* mRNA can help to induce flowering, but movement of the FT protein is much more crucial. In addition to FT proteins and RNA, other types of molecules, such as hormones and metabolites, have been postulated as long-distance floral signals (Turnbull, 2011; Dinant and Suárez-López, 2012).

## IS FT A PHLOEM-MOBILE TUBERIZATION SIGNAL?

Transmissible signals for flowering and tuberization are interchangeable. Tobacco scions induced to flower promote tuberization when grafted onto potato stocks kept under non-tuber-inducing conditions (Chailakhyan et al., 1981). When a rice Hd3a-GFP fusion is expressed in potato phloem, it can move across a graft junction to stolons and induce tuber formation (Navarro et al., 2011), suggesting that a similar protein exists in potato. Indeed, several *FT*-like genes have been identified in this species. One of them encodes StSP3D, which mainly affects flowering, and another encodes StSP6A, which induces tuber formation, a role similar to that of FT in flowering control (Navarro et al., 2011). The effect of StSP6A on tuberization is transmitted through grafts (Navarro et al., 2011). Altogether, these findings strongly suggest that StSP6A is probably a mobile tuberization signal.

**FIGURE 1:**
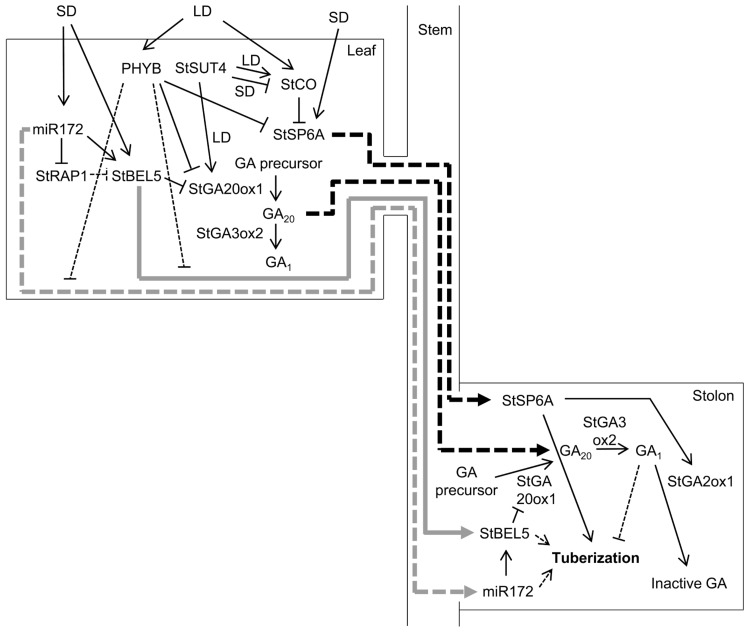
**Model for the regulation of tuber induction by phloem-mobile signals**. The main candidates for mobile signals are the StSP6A protein, two RNAs – *StBEL5* and miR172 – and GAs. The production, and possibly the movement, of these four factors is regulated by a complex genetic network. PHYB, StSUT4, and StCO repress tuberization in response to LDs. GAs also seem to act as repressors, whereas StSP6A and perhaps miR172 and StBEL5 act as tuberization promoters under inductive SD conditions. Under LDs, PHYB represses the expression of *StSP6A* and *StGA20ox1*, which encodes an enzyme that catalyzes the synthesis of GA_20_. PHYB up-regulates miR172 and StBEL5 in leaves and down-regulates them in stolons, which might result from a repression of *StBEL5* mRNA and miR172 movement from leaves to stolons. Under LDs, StSUT4 induces *StGA20ox1* and *StCO*, which represses *StSP6A*. Under SDs, StSUT4 inhibits *StCO*, relieving *StSP6A* repression. In addition to *StSP6A*, SDs up-regulate miR172 and *StBEL5*. miR172 induces *StBEL5*, probably through the repression of miR172 target genes, such as *StRAP1*, which would act as *StBEL5* inhibitors. StBEL5 represses *StGA20ox1* in a complex with POTH1. StGA3ox2 catalyzes the conversion of GA_20_ to GA_1_, an active GA. *StSP6A*, *StBEL5* mRNA, miR172, and GAs presumably translocate to stolons through the phloem. In the stolons, *StSP6A* promotes tuber development, at least in part through up-regulation of *StGA2ox1*, which converts active GAs into inactive forms. miR172 up-regulates *StBEL5*, which together with POTH1 down-regulates *StGA20ox1*, reducing the synthesis of active GAs, which repress tuber development. Under LDs, GA_20_ would move from leaves to stolons and would be converted to GA_1_, thus repressing tuber development. Under SDs, there would be less GA_20_ available and tuber development can occur. Thick gray arrows indicate RNA movement, and thick black arrows indicate protein or GA movement. Discontinuous lines indicate that movement or regulation has been suggested, but not demonstrated.

There are similarities, but also differences, in the regulation of FT genes. *StSP6A* is negatively regulated by StCO (**Figure 1**), a potato CO-like protein that represses tuberization under non-inductive LDs (Navarro et al., 2011; González-Schain et al., 2012). StCO does not seem to play a role under SDs (González-Schain et al., 2012). By contrast, *Arabidopsis* CO promotes *FT* transcription only under inductive photoperiods (Turck et al., 2008). In rice *Hd3a* is repressed or activated by the CO-like protein Hd1 under non-inductive or inductive conditions, respectively, and in addition *RFT1* is up-regulated and promotes flowering much later under non-inductive conditions (Tsuji et al., 2013). These differences stress the need to test hypotheses based on flowering-time models, rather than simply extrapolating them to tuberization. Demonstrations that StSP6A moves are therefore eagerly awaited.

Two additional FT family members from potato, *StTFL1* and *StSP5G*, might be related to the tuberization process. *StTFL1* mRNA levels are high in stolons before induction and decrease at early stages of tuber development. Overexpression of *StTFL1* causes an increase in the number of tubers produced (Guo et al., 2010), suggesting a role in tuber induction or development. The expression pattern of *StSP5G* suggests that this gene might play an opposite role to that of *StSP6A* in tuberization control (Navarro et al., 2011; Kloosterman et al., 2013), although a functional analysis of this gene has not been reported so far. Further analyses of *StTFL1* and *StSP5G* to determine their biological functions should be pursued, given that FT-related proteins affect other developmental processes aside from flowering and tuberization (Pin and Nilsson, 2012; Hiraoka et al., 2013). As many FT-like proteins are mobile, it would be worth testing *StTFL1* and *StSP5G* movement.

## *StBEL5* AND *POTH1* mRNAs AS PUTATIVE TRANSMISSIBLE SIGNALS

Two mRNAs have been proposed as long-distance signals regulating tuberization. StBEL5 and POTATO HOMEOBOX 1 (POTH1) are homeobox transcription factors that interact with each other (Chen et al., 2003). Overexpression of POTH1 increases the number of tubers produced relative to wild-type (WT) plants in *in vitro* tuberization assays (Rosin et al., 2003). Overexpression of StBEL5 enhances tuber formation under SDs and promotes tuberization under non-inductive LDs. *StBEL5* mRNA moves from overexpressing scions to WT stocks and movement correlates with increased tuber yield (Chen et al., 2003; Banerjee et al., 2006). Graft transmission of *POTH1* mRNA has also been shown (Mahajan et al., 2012). Transcription of *StBEL5* and *POTH1* in vascular cells (Banerjee et al., 2006; Mahajan et al., 2012) is consistent with movement of their transcripts through the phloem. Additional experimental approaches support translocation of *StBEL5* mRNA and have been previously reviewed (Hannapel, 2010).

However, there are numerous caveats to be aware of when interpreting the movement of *StBEL5* and *POTH1* RNAs, as well as their effects on tuberization. First, POTH1 has not been shown to affect tuber formation in soil-grown plants. Second, whether StBEL5 and/or POTH1 are required for tuber induction in WT plants has not been demonstrated, as only overexpression alters tuber induction or development. Third, RNA movement has been shown from overexpressing plants, but not from WT plants (Banerjee et al., 2006; Mahajan et al., 2012), and it has not been tested whether movement is required for tuberization. Fourth, POTH1-overexpressing plants exhibit dramatic alterations in the vasculature (Rosin et al., 2003; Mahajan et al., 2012). It is possible that the tuber phenotype of POTH1-overexpressing plants and graft transmission of *POTH1* mRNA are indirect consequences of these alterations. Fifth, both *POTH1* and *StBEL5* are transcribed in stolons, with an increase in *StBEL5* transcription at early stages of tuber formation (Banerjee et al., 2006; Mahajan et al., 2012), casting doubts on the need of movement from leaves. Finally, it has not been excluded that movement of StBEL5 and/or POTH1 proteins may occur.

Therefore, although *StBEL5* and *POTH1* RNAs are able to move, further research is needed to demonstrate whether this has any biological relevance. This can be addressed by simultaneously silencing *StBEL5* and *POTH1* or several *StBEL* paralogs, which have been proposed to act redundantly (Chen et al., 2003). Whether the StBEL5 protein moves should also be tested.

## miR172 AFFECTS TUBERIZATION IN A GRAFT-TRANSMISSIBLE MANNER

To date, miR172, which regulates flowering in several species, is the only microRNA (miRNA) shown to affect tuber induction (Martin et al., 2009; Zhu and Helliwell, 2011). The effect of miR172 in potato has been reported in overexpressing plants, which form tubers under LDs, tuberize early under SDs and show up-regulation of *StBEL5*. Inactivation would help to confirm if miR172 is required for tuberization control.

There is growing evidence that small RNAs, including short interfering RNAs (siRNAs) and miRNAs, move cell-to-cell and systemically (Himber et al., 2003; Yoo et al., 2004; Lin et al., 2008; Pant et al., 2008; Chitwood et al., 2009; Carlsbecker et al., 2010; Dunoyer et al., 2010; Molnar et al., 2010). The effect of miR172 overexpression is graft transmissible, suggesting that this miRNA regulates long-distance signals that control tuberization or, alternatively, that miR172 itself is a mobile signal. In grafting experiments, miR172-overexpressing scions accelerated tuberization of WT stocks, but the reciprocal graft combination did not tuberize early. The simplest interpretation is that miR172 is required in aerial organs, rather than in stolons, to promote tuberization. However, increases of miR172 levels in stolons correlate with tuber induction, while changes in leaves do not (Martin et al., 2009). At least two hypotheses can explain this apparent contradiction: (1) overexpression of miR172 in stocks might not be sufficient to counteract tuber-inhibiting signals derived from WT scions; and (2) factors required for miR172 processing might be present or active in leaves but not in stolons. Detection of miR172 in potato phloem cells and phloem exudates of several species, as well as graft transmission in *Nicotiana benthamiana*, is consistent with the notion of this miRNA being mobile (Buhtz et al., 2008, 2010; Martin et al., 2009; Kasai et al., 2010; Varkonyi-Gasic et al., 2010). In addition to its putative role as a systemic signal, it has been proposed that miR172 might participate in cell-to-cell communication (Abelenda et al., 2011; Marín-González and Suárez-López, 2012). Given the potential of miRNAs to act as transmissible signals, it will be worth studying whether miR172 moves.

## ROLE OF GIBBERELLINS IN TUBERIZATION

The plant hormones gibberellins (GAs) are present in phloem sap and seem to act as florigenic molecules in some species (Eriksson et al., 2006; King et al., 2006, 2008). The last steps in the biosynthesis of active GAs are catalyzed by GA 20-oxidase (GA20ox) and GA 3-oxidase (GA3ox). Biologically active GAs, including GA_1_, GA_3_, and GA_4_, are inactivated by GA 2-oxidase (GA2ox) enzymes (Hedden and Thomas, 2012).

Gibberellins are involved in the control of tuber induction or development. Different observations have led to the assumption that GAs inhibit tuberization under LDs. Tuberization would take place when GA levels decrease in response to SDs (Rodríguez-Falcón et al., 2006). This decrease seems necessary to arrest longitudinal stolon growth and allow stolon swelling (Jackson, 1999). But are high GA levels really required to repress tuber induction under LDs? Silencing of a potato GA20ox (StGA20ox1) and manipulation of the levels of a GA3ox (StGA3ox2) do not induce tuberization under LDs (Carrera et al., 2000; Bou-Torrent et al., 2011). In addition, a GA2ox, StGA2ox1, affects tuberization *in vitro*, but not in soil-grown plants (Kloosterman et al., 2007), leading to the conclusion that *StGA2ox1* is a tuber-identity gene rather than a regulator of tuber induction. Local up-regulation of *StGA2ox1* in stolons by StSP6A (Navarro et al., 2011) is consistent with this interpretation.

Moreover, the expression patterns of several GA biosynthetic enzymes and the phenotypes of plants with altered levels of these enzymes do not always fit with the hypothesis of GAs repressing tuberization. For example, although *StGA3ox2* is down-regulated at the initiation of tuber development, *StGA20ox1* and *StGA20ox3* are up-regulated (Kloosterman et al., 2007). Both StGA20ox1-silenced lines and plants overexpressing StGA3ox2 tuberize earlier than WT plants under SDs, despite showing opposite changes of GA_1_ levels (Carrera et al., 2000; Bou-Torrent et al., 2011). As GA biosynthesis involves feedback and feedforward regulations (Hedden and Thomas, 2012), some of these contradictions can be explained through negative feedback regulation of *StGA20ox* genes by active GAs, but this still has to be demonstrated.

To explain some of these conflicting results, it has recently been proposed that GA_20_ – the immediate precursor of GA_1_ – would be mobile, whereas GA_1_ would not. In StGA3ox2-overexpressing plants, increased conversion of GA_20_ to GA_1_ in aerial parts would reduce the amount of GA_20_ transported to stolons, resulting in low levels of GA_1_ in stolons and early tuberization (Bou-Torrent et al., 2011). This interesting hypothesis fits well with some observations. However, as *StGA20ox* genes are expressed in stolons (Carrera et al., 1999), GA_20_ is expected to be synthesized here. StGA3ox2-overexpressing plants would then have increased conversion of GA_20_ to GA_1_ also in stolons, which should repress tuberization. Localized silencing of StGA20ox1 and StGA3ox2 in leaves and stolons and grafting experiments using plants with altered levels of these enzymes would help to elucidate the role of GA_20_ and GA_1_. It will also be necessary to test GA_20_ movement in potato plants and whether movement is required to prevent tuberization. More work is also needed to determine whether GAs play a role in tuber induction or they regulate tuber development by preventing stolons from being competent to respond to leaf-derived inductive signals. Nowadays it cannot be excluded that GAs perform both functions.

## SUCROSE AND OTHER PUTATIVE LONG-RANGE SIGNALING MOLECULES

Sucrose is a metabolite, a source of energy and a signaling molecule and it has been proposed as a transmissible substance for tuberization and flowering (Sheen et al., 1999; Suárez-López, 2005; Ruan, 2012). Transcripts of sucrose transporters are phloem mobile in several species, including potato, which suggests a possible signaling role for these RNAs (Liesche et al., 2011). A potato sucrose transporter, StSUT4, is involved in flowering and tuberization control. Inhibition of StSUT4 induces tuberization under LDs. Graft transmission of this phenotype, together with an increase in sucrose export from leaves of StSUT4-silenced plants, suggest a role for StSUT4 in long-distance signaling at least in part via source to sink carbon flux (Chincinska et al., 2008). In addition, StSUT4 regulates the production of putative long-distance signals, such as StSP6A and probably GAs (Chincinska et al., 2008, 2013).

There is additional evidence of a link between sucrose and GAs during tuberization. *In vitro* treatment with high sucrose concentrations, which induces tuber formation, reduces endogenous GA_1_ levels in stolons before tuber initiation (Xu et al., 1998). Exogenous GA treatment, conversely, up-regulates StSUT4 (Chincinska et al., 2008). Altogether these observations indicate a complex interplay between GAs and sucrose during tuber induction or development. Understanding the different roles that sucrose plays in tuber formation, as a starch precursor, energy source and signal, deserves further attention.

Other molecules, such as metabolites, hormones, and peptides have the potential to act as mobile signals, but their roles in tuberization are not yet clear (Jackson, 1999; Fernie and Willmitzer, 2001; Dinant and Suárez-López, 2012). Grafting of tomato mutants onto potato stocks has been proposed as a strategy to elucidate the role of hormones in long-distance signaling, although the results so far point to effects on assimilate distribution rather than on signaling pathways (Peres et al., 2005).

## THE ROLE OF PHYTOCHROME B IN REGULATING MOBILE SIGNALS

The photoreceptor phytochrome B (PHYB) plays an interesting role in the control of tuber induction, as it affects several putative systemic tuberization molecules. Grafting experiments using PHYB-silenced plants, which tuberize under LDs, led to the proposal that PHYB induces a mobile tuberization repressor(Jackson et al., 1998). However, these plants show increased levels of *StSP6A* mRNA and reduced levels of *StBEL5* mRNA and miR172 in leaves, as well as increased levels of these three RNAs in stolons (Martin et al., 2009; Navarro et al., 2011), indicating that PHYB inhibits the expression and/or movement of tuber-inducing molecules. Probably positive and negative regulators of tuberization respond to light signals through the action of PHYB.

Several effects of PHYB on plant development are mediated by GAs (Lau and Deng, 2010). In potato, PHYB affects GA synthesis or signaling, as *StGA20ox1* mRNA abundance is increased in PHYB-silenced plants, which show several phenotypes characteristic of alterations in GA homeostasis (Jackson et al., 2000). In addition, StSUT4 probably mediates some effects of PHYB on plant development (Chincinska et al., 2008). How PHYB regulates all these genes is not known. PHYB controls long-distance regulation of other processes, pointing to a general role of PHYB in systemic signaling (Griebel and Zeier, 2008; Suzuki et al., 2011).

## CONCLUSION AND FUTURE CHALLENGES

We have recently witnessed substantial advances in our knowledge of potato tuber induction. Although the identity of mobile tuberization molecules is yet unknown, they are probably similar to flowering signals. Several good candidates have been proposed (**Figure 1**). Further research should test whether they act as genuine systemic tuberization signals.

Long-distance communication involves the production of signals, but also requires phloem loading, transport and unloading, as well as the response of target tissues to the translocated signals. Once the chemical nature of the signals is established, dissecting all these processes will be easier. The availability of the potato genome sequence (Xu et al., 2011) will facilitate these tasks. Interspecific grafting and experimental approaches used in other species, such as analyses of phloem sap composition, visualization of reporters fused to putatively mobile proteins and strategies to disrupt intercellular signaling, can be employed to address these questions.

## Conflict of Interest Statement

The author declares that the research was conducted in the absence of any commercial or financial relationships that could be construed as a potential conflict of interest.
